# Physicochemical characterization and cancer cell antiproliferative effect of silver-doped magnesia nanoparticles

**DOI:** 10.1016/j.heliyon.2023.e15560

**Published:** 2023-04-17

**Authors:** Mohamed Qasim Al-Fahdawi, Ahmed Faris Aldoghachi, Fatah H. Alhassan, Faris A.J. Al-Doghachi, Hussah Abdullah Alshwyeh, Abdullah Rasedee, Sulaiman Mohammed Alnasser, Mothanna Sadiq Al-Qubaisi, Wisam Nabeel Ibrahim

**Affiliations:** a Institute of Bioscience, Universiti Putra Malaysia, 43400 UPM Serdang, Selangor, Malaysia; b Faculty of Medicine and Health Sciences, University Putra Malaysia, UPM, Serdang, 43300, Malaysia; c Faculty of Medicine and Health Sciences, Universiti Tunku Abdul Rahman, Cheras, 43000, Malaysia; d Department of Applied Chemistry and Technology, College of Science and Arts, Alkamel University of Jeddah, Jeddah, 21589, Saudi Arabia; e Department of Nanoscience and Nanotechnology, Africa City of Technology, Khartoum Bahari, Khartoum, Sudan; f Department of Chemistry, Faculty of Science, University of Basra, Basra, Iraq; g Department of Biology, College of Science, Imam Abdulrahman Bin Faisal University, Dammam, 31441, Saudi Arabia; h Basic & Applied Scientific Research Center, College of Science, Imam Abdulrahman Bin Faisal University, Dammam, 31441, Saudi Arabia; i Department of Veterinary Laboratory Diagnosis, Faculty of Veterinary Medicine, Universiti Putra Malaysia, 43400 UPM Serdang, Selangor, Malaysia; j Department of Pharmacology and Toxicology, Unaizah College of Pharmacy, Qassim University, Saudi Arabia; k Department of Biomedical Science, College of Health Sciences, QU Health, Qatar University, Doha, Qatar

**Keywords:** Ag/MgO nanoparticles, HT29 cell, A549 cell, Antiproliferative, Apoptosis

## Abstract

Silver-doped magnesia nanoparticles (Ag/MgO) were synthesized using the precipitation method and characterized by various techniques such as X-ray diffraction (XRD), Fourier transform infrared spectroscopy (FT-IR), thermal gravimetric analysis (TGA), Brunner-Emmett-Teller (BET) surface area measurements, and dispersive X-ray spectroscopy (EDX). The morphology of Ag/MgO nanoparticles was determined by transmission and scanning electron microscopy, which revealed cuboidal shaped nanoparticles with sizes ranging from 31 to 68 nm and an average size of 43.5 ± 10.6 nm. The anticancer effects of Ag/MgO nanoparticles were evaluated on human colorectal (HT29) and lung adenocarcinoma (A549) cell lines, and their caspase-3, -8, and -9 activities, as well as Bcl-2, Bax, p53, cytochrome C protein expressions were estimated. Ag/MgO nanoparticles showed selective toxicity towards HT29 and A549 cells while remaining relatively innocuous towards the normal human colorectal, CCD-18Co, and lung, MRC-5 cells. The IC_50_ values of Ag/MgO nanoparticles on the HT29 and A549 cells were found to be 90.2 ± 2.6 and 85.0 ± 3.5 μg/mL, respectively. The Ag/MgO nanoparticles upregulated caspase-3 and -9 activities, downregulated Bcl-2, upregulated Bax and p53 protein expressions in the cancer cells. The morphology of the Ag/MgO nanoparticle treated HT29 and A549 cells was typical of apoptosis, with cell detachment, shrinkage, and membrane blebbing. The results suggest that Ag/MgO nanoparticles induce apoptosis in cancer cells and exhibit potential as a promising anticancer agent.

## 1 Introduction

Nanoparticles are able to penetrate the body through dermal deposition, injection into the bloodstream, inhalation and ingestion, and can be transported through the blood to various organs and systems of the body by numerous routes [1,2]. Once inside the human body, nanoparticles can enter the cell through a number of mechanisms. After reaching the intracellular environment, the nanoparticles can interact with the genetic material due to their random permanence inside the nucleus after mitosis, or through the nuclear pores if they are small diameter nanoparticles [3,4]. These particles can damage organelles and other cellular components and when they are inside the nucleus they can interact directly with the DNA to induce damage to the genetic material [4,5].

Nanotechnology offers the way for targeted and selective treatment of tumor cells. Routine chemotherapy uses drugs that are known to have the ability to successfully kill tumor cells [6,7]. But these cytotoxic drugs kill healthy cells too, causing side effects such as neuropathy, hair loss, and impaired immune function. Nanotechnology offers the way to treatments selectively in tumor cells, which can be used as drug carriers, being directed directly to target cells [6,7]. For this purpose, cell culture has been widely used to perform in vitro cytotoxicity tests in the evaluation of potential antineoplastic agents and the safety of compounds such as cosmetics, drugs, pesticides, food agents and industrial chemicals [8,9].

Noble metal nanoparticles, such as gold and silver nanospheres, are gaining utility in clinical applications because they can be engineered to exhibit specific therapeutic properties [[10], [11], [12], [13]]. The surface of metal nanoparticles can be modified by conjugation with therapeutic molecules [14] such as antibodies [15]. Metal nanoparticles loaded with therapeutic molecules are stable, exhibit low toxicity, and have the potential for tissue and organ targeting, making them effective and desirable as therapeutic compounds.

Nanoparticles can be modified to provide unique physicochemical properties by altering their core sizes and shapes [[16], [17], [18]]. One of the methods used to physiochemically modify the nanoparticles is through the oxidation and reduction reactions using sodium borohydride [19,20] and magnesium oxide [21,22].

Silver nanoparticles have been shown to possess numerous therapeutic properties including anticancer, antibacterial, and larvicidal properties [23]. These particles can be synthesized by reducing silver nitrate into nanoparticulated elemental silver [[24], [25], [26]] in various shapes, including rods, spheres, triangles, octagons, and hexagons [27]. In the presence of a reducing agent and under acidic pH, silver nitrate can grow into flat triangular silver nanocrystals [[28], [29], [30], [31]]. When excited by light of specific wavelengths, the conduction electrons on the surfaces of silver nanoparticles undergo collective oscillation, which can be detected by the surface plasmon resonance and as specific peaks on the absorption spectrum. The surface plasmon resonance showed that the silver nanoparticles are within the near-infrared spectrum, thus, they can be laser-heated to improve their anticancer effects in tumors [[32], [33], [34]]. Exposure to silver nanoparticles can compromise, through the formation of ROS, the cell signaling cascade that controls cell proliferation, inflammatory processes mediated by transcription factors NFkB and AP1, and cell death by apoptosis [[35], [36], [37], [38]].

In this study, silver-doped magnesia (Ag/MgO) nanoparticles were developed as a potential anti-cancer formulation for human colorectal and lung adenocarcinomas.

## 2 Materials and methods

### 2.1 Chemicals and reagents

In our study, we utilized various chemicals, including Dulbecco’s modified Eagle’s medium (DMEM), fetal bovine serum (FBS), phosphate-buffered saline (PBS), trypsin/ethylenediaminetetraacetic acid (EDTA) (Invitrogen, Carlsbad CA, USA), Dimethylsulfoxide (DMSO), and 3-(4,5-dimethylthiazol-2-yl)-2,5-diphenyltetrazolium bromide (MTT). Additionally, we employed diphenylamine (DPA) reagent, which comprises of glacial acetic acid (100 mL), 1.5 g diphenylamine (1.5 g), 1.5 mL concentrated sulfuric (1.5 mL), and acetaldehyde (16 mg/mL) (stock), as well as trypan blue dye (Perth, WA, Australia) in our experiments.

### 2.2 Preparation of Ag/MgO nanoparticles

To prepare the Ag/MgO nanoparticles, we utilized the precipitation method, as outlined in a previous study [39]. Firstly, we prepared magnesium oxide (MgO) using a solution of 0.1 M aqueous Mg(NO_3_)_2_.6H_2_O (Merck; >99.0%) and 1 M K_2_CO_3_ (Merck; >99.7%) as precipitants. The resulting precipitate was filtered, washed with hot water, and dried at 120 °C for 12 h. To remove CO_2_, the precipitate was pre-calcined for 5 h at 500 °C in the air, pressed into discs at 600 kg/m^2^ (Hydraulic for KBr disc), and then calcined for 20 h at 1150 °C.

**Fig. 1:**
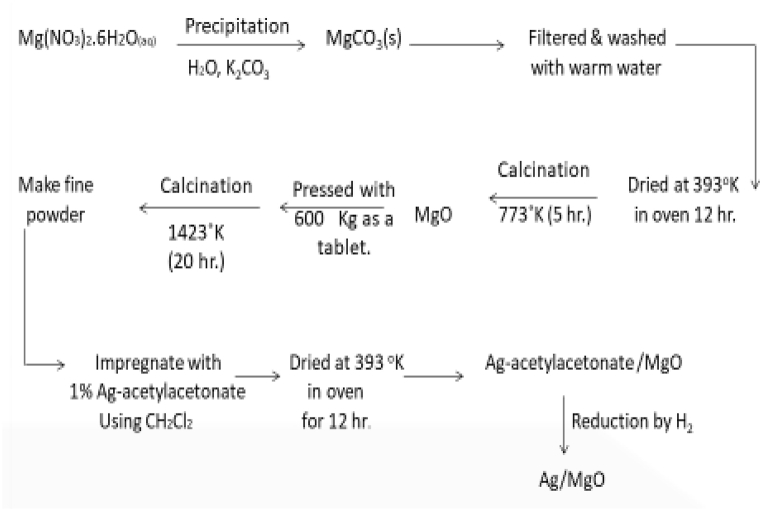
Flow chart presentation of the silver-doped magnesia nanoparticles (Ag/MgO) preparation using the precipitation method.

Next, the sample was impregnated with 5% Ag [Ag(C_5_H_7_O_2_)_2_.H_2_O (Acros Chemicals, >99%)], and the impregnated sample was dissolved in dichloromethane for 5 h to produce Ag(acac)2/MgO. After impregnation in air, the sample was dried at 120 °C for 12 h, crushed into powder, and sieved (pore size: 250 μm) to obtain particles of 80–150 or 150–250 μm in diameter. The Ag^2+^ phase nanoparticles were then reduced to the metal Ag° phase using 5% H_2_/Ar, resulting in the final product, the Ag/MgO nanoparticles. For clarity, a schematic representation of the Ag/MgO nanoparticle preparation is provided in Fig. 1.

### 2.3 Physicochemical properties of nanoparticles

#### 2.3.1 Scanning electron microscopy and energy dispersive X-ray spectroscopy

To investigate the elemental composition and morphology of the Ag/MgO nanoparticle powder, we used energy dispersive X-ray spectroscopy (EDX) and scanning electron microscopy (SEM, Model LEO 1450VP, LEO Electron Microscopy Ltd Cambridge, UK) at an accelerating voltage of 30 kV. Prior to analysis, the samples were degassed overnight at 100 °C in an evacuated chamber. To prepare the samples for SEM analysis, the dried powder was spread on double-sided conductive tapes and adhered to a specimen stub. Subsequently, the samples were subjected to scanning electron microscopy to obtain images and analyze their morphology. Additionally, EDX was utilized to determine the elemental composition of the nanoparticle powder.

#### 2.3.2 Transmission electron microscopy

In our study, we investigated the crystalline ultrastructure of the Ag/MgO nanoparticles using transmission electron microscopy (TEM) (Hitachi H-7100, Japan). TEM is a high-resolution microscopy technique that is widely used to study the structure and properties of nanomaterials. To prepare the samples for TEM analysis, we suspended the Ag/MgO nanoparticle powder in deionized water and placed a drop onto carbon-covered copper grids. We then allowed the suspension to dry at room temperature before subjecting it to TEM analysis.

The use of carbon-covered copper grids in TEM analysis allows for the nanoparticles to be immobilized in a stable and uniform manner, facilitating accurate imaging and analysis. Once the samples were prepared, we examined the crystal structure of the Ag/MgO nanoparticles at high magnification to determine their size, shape, and composition. By analyzing the images obtained from TEM, we were able to determine the crystalline ultrastructure of the nanoparticles and gain insight into their physical and chemical properties.

#### 2.3.3 X-ray diffraction

The Ag/MgO nanoparticle powder underwent characterization using X-ray diffraction (Shimadzu diffractometer model XRD 6000, Japan). During the analysis, Cu-Kα radiation was generated at ambient temperature by a Philips glass diffraction X-ray tube with a broad focus of 2.7 kW type. We determined the crystallite size D of the sample using Debye-Scherrer's relationship [40,41] which is a widely accepted method for determining the crystal size of nanoparticles This was done by using the following formula:

$$\text{T }=\frac{0.9\lambda }{\beta \text{cos}\theta }$$

where T is the crystallite size, K is the Scherrer constant (0.89), λ is the wavelength of the X-rays used, β is the full width at half maximum (FWHM) of the peak, and θ is the diffraction angle.

#### 2.3.4 Fourier transform infrared

o obtain the Fourier transform infrared (FTIR) spectra for the Ag/MgO nanoparticle powder, we used approximately 1% of the sample in 200 mg spectroscopic-grade potassium bromide (KBr). The spectra were determined using the Smart Orbit spectrometer (Thermo Nicolet Nexus, Shelton, USA) over the range of 400–4000 cm^−1^ and under 10-ton pressure.

FTIR spectroscopy is a powerful technique for analyzing the chemical composition and molecular structure of materials. In this study, we utilized FTIR spectroscopy to analyze the Ag/MgO nanoparticle powder and obtain information about the chemical bonds and functional groups present in the sample. The use of KBr as a matrix for FTIR analysis allows for the sample to be uniformly dispersed and provides a stable environment for analysis. The pressure applied during analysis helps to ensure consistent and accurate measurements.

#### 2.3.5 Thermogravimetric analysis

In order to determine the thermal strength of the Ag/MgO nanoparticles, we used the Mettler Toledo TG-SDTA apparatus (Switzerland) with Pt crucibles and a Pt/Pt– Rh thermocouple. The analysis was carried out under a purge nitrogen gas flow rate of 30 mL min^−1^. The heating rate used for the analysis was 10 °C min ^−1^, and the temperature range measured was from room temperature to 1000 °C.

Thermal analysis is a powerful technique for characterizing the thermal stability of materials, including nanoparticles. In this study, we utilized the TG-SDTA apparatus to analyze the Ag/MgO nanoparticles and obtain information about their thermal stability under different conditions. The use of Pt crucibles and a Pt/Pt– Rh thermocouple helps to ensure accurate measurements of temperature and thermal properties.

#### 2.3.6 Brunauer, Emmett, and Teller analysis

To determine the total surface area of the Ag/MgO nanoparticles, we used the nitrogen adsorption-desorption analysis technique with a Surfer analyzer (Milan, Italy). The analysis was carried out at a temperature of −196 °C, and nitrogen gas was used as the adsorbate.

Nitrogen adsorption-desorption analysis is a widely used technique for determining the surface area and porosity of materials, including nanoparticles. The technique involves measuring the adsorption and desorption of a gas onto the surface of the sample at different temperatures and pressures. The data obtained can be used to calculate the specific surface area of the sample and provide information about its porosity.

#### 2.3.7 Hydrodynamic size and zeta potential

The zeta potential and hydrodynamic size of the Ag/MgO nanoparticle dispersion were determined using the ZetaSizer Nano ZS (Malvern Instruments Ltd, Malvern, UK) equipped with dynamic light scattering. For this analysis, we used a nanoparticle dispersion containing 1 μg of nanoparticles in 1 mL of ultra-deionized water.

Dynamic light scattering (DLS) is a widely used technique for characterizing the size distribution of nanoparticles in solution. The technique involves measuring the movement of nanoparticles under Brownian motion and calculating their hydrodynamic size based on the diffusion coefficient. Additionally, the zeta potential is measured using the electrophoretic mobility of the nanoparticles in the dispersion.

### 2.4 Cell culture

In this study, we utilized four human cell lines from the American Type Culture Collection (ATCC) in Rockville, MD, USA. These cell lines included virus-negative human colorectal adenocarcinoma (HT29), human lung adenocarcinoma (A549), normal human colon (CCD-18Co), and normal lung (MRC-5) cells.

All of the cell lines were cultured in DMEM medium (Sigma Aldrich, USA) at 37 °C under 5% CO_2_. The medium was supplemented with 10% fetal bovine serum (FBS) and 1% penicillin (100 U/mL) (Isocillin, Aventis, Germany). The use of FBS in cell culture is common as it provides essential nutrients and growth factors necessary for cell growth and survival. Additionally, the addition of penicillin helps to prevent contamination in the cell culture.

The choice of cell lines used in this study is important, as it can impact the relevance and applicability of the results to the disease being studied. By using both normal and cancerous cell lines from different organs, we aimed to investigate the potential differential effects of the Ag/MgO nanoparticles on different cell types.

### 2.5 3-(4,5-dimethylthiazol-2-yl)-2,5-diphenyltetrazolium bromide assay

The study involved mixing the Ag/MgO nanoparticles thoroughly in DMEM medium (Sigma Aldrich, USA) supplemented with 10% heat-inactivated FBS. To obtain the nanoparticle colloidal suspension, the ultrasound method was used [40,41]. 200 μL of 5 × 10^3 cells/mL cancer and 5 × 10^5 cells/mL normal cell suspensions were seeded into the designated wells of a 96-well cell culture plate. After aspiration of the medium, 200 μL of Ag/MgO nanoparticles in the medium at concentrations ranging from 1.56 to 100 μg/mL (synthesized in-house) and chemotherapeutic agents (oxaliplatin for HT29 and CCD-18Co cells, and paclitaxel for A549 and MRC-5 cells) at concentrations ranging from 0.156 to 10.0 μg/mL (Sigma Aldrich, USA) were added to the respective wells, with one row of wells serving as the non-treatment control. The plate was incubated at 37 °C under 5% CO_2_ for 24 h. After removing the medium, the cells were washed three times with PBS buffer, and then 200 μL of fresh medium containing 5 mg/mL 3-(4,5-dimethylthiazol-2-yl)-2,5-diphenyltetrazolium bromide (MTT) solution (Sigma Aldrich, USA) was added to each well. The plate was further incubated at 37 °C under 5% CO_2_ for 4–6 h, followed by careful removal of the medium and addition of 200 μL DMSO (Sigma Aldrich, USA) to each well to dissolve the formazan crystals. The optical density of the samples was measured at 570 nm using an automated spectrophotometric EL 340 multiplate reader (Bio-Tek Instruments Inc., USA). The cell viability was calculated as follows:

$$\text{Cell viability }\left(\%\right)=\frac{{\text{OD}}_{\text{Treatment}}}{{\text{OD}}_{\text{Nontreatment}}}\times 100$$

where OD is the optical density.

The 24-h IC_50_ values (50% cell growth inhibition concentration) were obtained by generating dose-response curves for each cell line.

### 2.6 Caspases

The study evaluated the impact of 12- and 24-h treatments with different concentrations (3.125, 6.25, and 12.5 μg/mL) of Ag/MgO nanoparticles on the caspase-3, -8, and -9 activities of HT29 and A549 cells. The caspase activities were measured using commercially available colorimetric assay kits (Promega, Madison, WI, USA) according to the manufacturer's instructions.

### 2.7 Quantification of *Bax*, *p53*, *Bcl-2* and cytochrome C protein assay

The study investigated the effects of different concentrations (3.125, 6.25, and 12.5 μg/mL) of Ag/MgO nanoparticles on the expression levels of p53, Bax, Bcl-2, and cytochrome C proteins in HT29 and A549 cells. The treatment duration was 12 and 24 h, and the protein expression was determined using ELISA assay kits from R&D Systems, MN, USA. The ELISA assay kits were chosen due to their high sensitivity and specificity in detecting target proteins. The p53, Bax, Bcl-2, and cytochrome C proteins play critical roles in apoptosis and are often used as biomarkers for evaluating the efficacy of anticancer agents. The results of this study could provide insights into the mechanisms underlying the anticancer effects of Ag/MgO nanoparticles.

### 2.8 Microscopic examination of cell morphology

The experimental setup involved seeding 200 μL of either 1 × 10^4^ HT29 or A549 cell suspension into the respective wells of a 6-well plate. After treatment with the IC_50_ concentrations of Ag/MgO nanoparticles, the cells were subjected to morphological examinations using an inverted microscope. The morphological changes were analyzed and recorded to evaluate the impact of the nanoparticles on the cells' structure and appearance.

### 2.9 Statistical analysis

Unless otherwise specified, all experiments in this study were conducted in triplicate to ensure the reliability of the results. The data obtained from the experiments were analyzed using the Minitab statistical software (Minitab Inc., State College, PA), and the values are presented as means ± standard deviation (SD) to indicate the degree of variability in the data. To determine if there were any statistically significant differences among the means of the various treatment groups, we used one-way analysis of variance (ANOVA) followed by Dunnett's multiple comparison tests. A p-value of less than 0.05 was considered to be statistically significant, unless otherwise specified.

## 3 Results

### 3.1 Material characterization

#### 3.1.1 Morphological and elemental characterization of silver-doped magnesia nanoparticles

##### 3.1.1.1 Scanning electron microscopy and energy dispersive X-ray spectroscopy

**Fig. 2:**
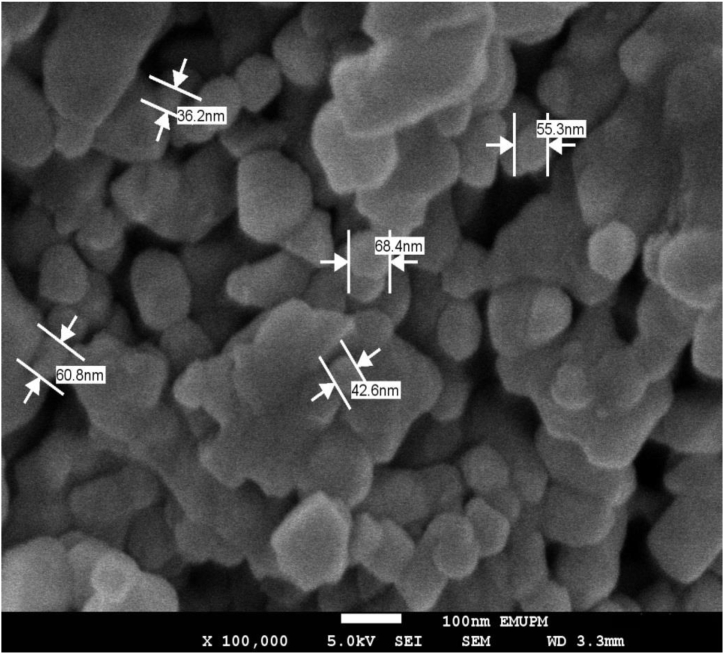
Scanning electron microscopic structure of Ag/MgO nanoparticles. The nanoparticles are well-dispersed with sizes ranging from 41 to 65 nm.

**Fig. 3:**
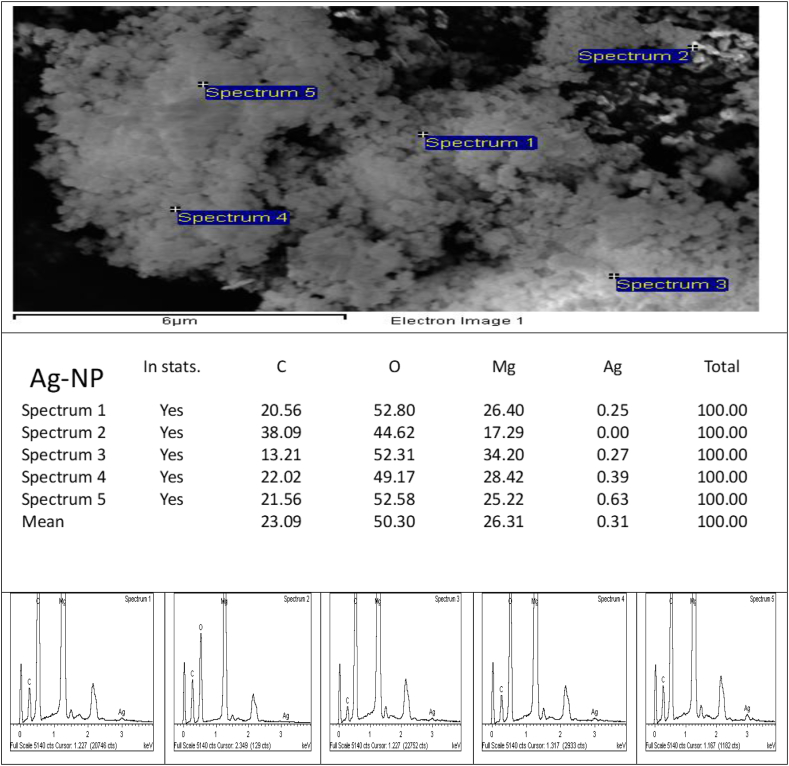
Energy dispersive X-ray spectroscopy of Ag/MgO nanoparticles. Cross-sectional SEM-EDX mapping demonstrates the fine-grain layer of powdered Ag/MgO nanoparticles comprised mostly of Ag/MgO. There is no evidence of pure Ag or MgO.

The pure Ag/MgO nanoparticle samples can be observed through SEM and EDX micrographs, which are displayed in Fig. 2, Fig. 3 correspondingly. These imaging techniques provide valuable insights into the morphology and elemental composition of the nanoparticles. The Ag/MgO nanoparticles are seen to form large clusters, signifying their propensity to aggregate. This aggregation behaviour is a consequence of the strong interactions among the particles, which could be attributed to van der Waals forces, electrostatic forces, or other interparticle forces. The aggregation of these nanoparticles may have a significant impact on their properties and potential applications, making it an important aspect to consider in future research and development [40,41].

##### 3.1.1.2 Transmission electron microscopy

**Fig. 4:**
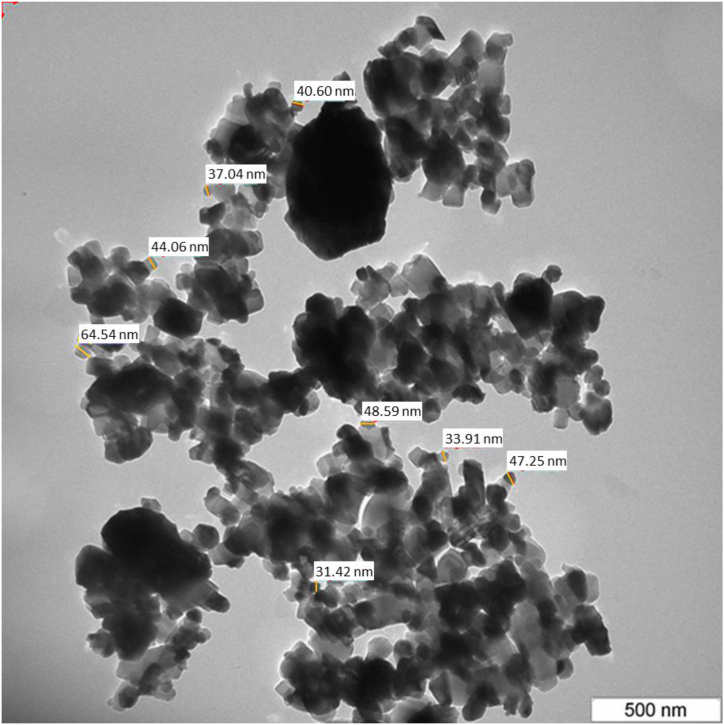
Transmission Electron microscopic structure of Ag/MgO nanoparticles. TEM analysis is necessary to determine the material differences in the Ag/MgO nanoparticles.

Fig. 4 presents the TEM micrograph of Ag/MgO nanoparticles, revealing valuable information about their morphology and size distribution. The particles are characterized by a cuboidal shape and sizes ranging from 31 to 64 nm. The average size of these nanoparticles is found to be 43.5 ± 10.6 nm, indicating a relatively broad size distribution.

A notable observation from the TEM micrograph is the aggregation of most Ag/MgO nanoparticles. This phenomenon is primarily attributed to the strong electrostatic interactions among the particles. Electrostatic forces can arise from the surface charges of the nanoparticles or the presence of charged species in the surrounding environment. The aggregation behaviour may have a significant impact on the nanoparticles' properties, such as their surface area, reactivity, and potential applications.

#### 3.1.2 Crystallinity

**Fig. 5:**
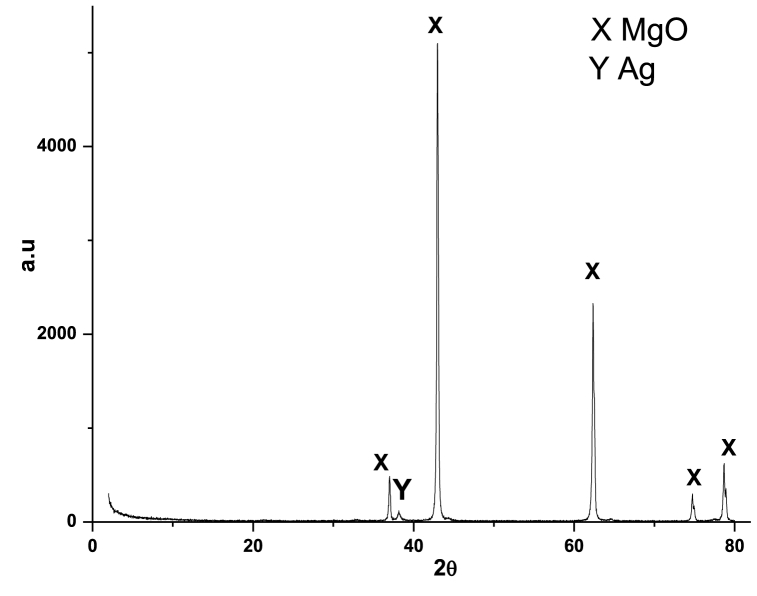
X-ray diffraction (XRD) patterns of Ag/MgO nanoparticles. The data confirm the formation of the Ag/MgO nanoparticles, their definitive structure, and phase purity identification.

The X-ray diffraction (XRD) powder pattern for Ag/MgO nanoparticles, calcined at 1150 °C for 20 h, is displayed in Fig. 5. The primary phase of the nanoparticles is cuboidal, as evidenced by the 2θ values of 36.9° (111), 62.3° (220), 74.8° (311), and 78.6° (222) (JSPDS file no.: 01-081-1545). The intensity ratio of the 200 to 111 plane, which is more than 10 times higher, suggests that the Ag/MgO nanostructures are predominantly characterized by the 200 plane. The distribution of magnesium oxide within the Ag/MgO nanoparticles enhances the stability of their cuboidal phase, resulting in a high Brunauer-Emmett-Teller (BET) surface area [42]. The crystal size of the Ag/MgO nanoparticles, as calculated using the Debye-Scherrer equation, was 39.2 nm. The BET-specific surface area (S_BET), pore volume, and pore radius of the Ag/MgO nanoparticles measured 14.78 m^2^/g, 0.23 cm^3^/g, and 10.3 Å, respectively.

#### 3.1.3 Infrared absorption bands

**Fig. 6:**
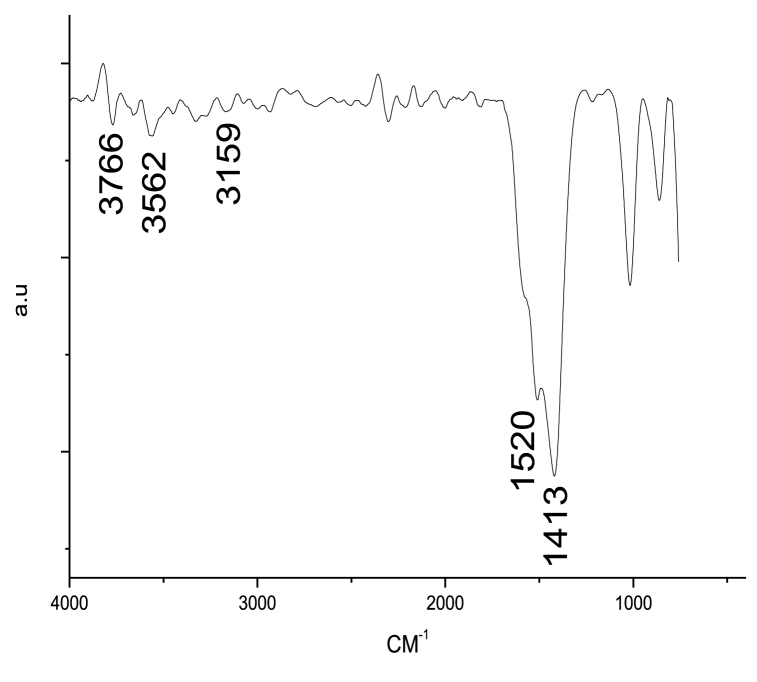
Fourier Transform Infrared (FTIR) spectroscopy analysis displaying the vibrational spectrum of Ag/MgO nanoparticles.

Fig. 6 illustrates the Fourier Transform Infrared (FT-IR) spectrum of the Ag/MgO nanoparticles, which provides information about the functional groups and chemical bonds present in the material. A weak vibrational band at 3562 cm^−1^ suggests that the Ag/MgO nanoparticle sample has a low moisture content. This observation is essential for understanding the sample's stability and potential applications, as moisture could affect the performance and properties of the nanoparticles.

The peaks appearing at approximately 3159 and 1520 cm^−1^ correspond to the stretching and bending modes of hydroxyl groups within the nanoparticles, respectively. The presence of these hydroxyl groups on the surfaces of Ag/MgO nanoparticles indicates that they possess Brønsted acid-active sites. These sites may play a crucial role in catalytic applications, as they can participate in acid-catalyzed reactions and promote specific reaction pathways.

The band at 3766 cm^−1^ is associated with the stretching vibration, while the band at 1413 cm^−1^ relates to the –OH bending in the bonds of the Ag/MgO nanoparticles. These spectral features provide additional insights into the molecular structure and interactions within the material. Understanding the bonding characteristics and functional groups present in the Ag/MgO nanoparticles is essential for tailoring their properties for targeted applications and optimizing their synthesis processes.

#### 3.1.4 Thermogravimetric analysis

**Fig. 7:**
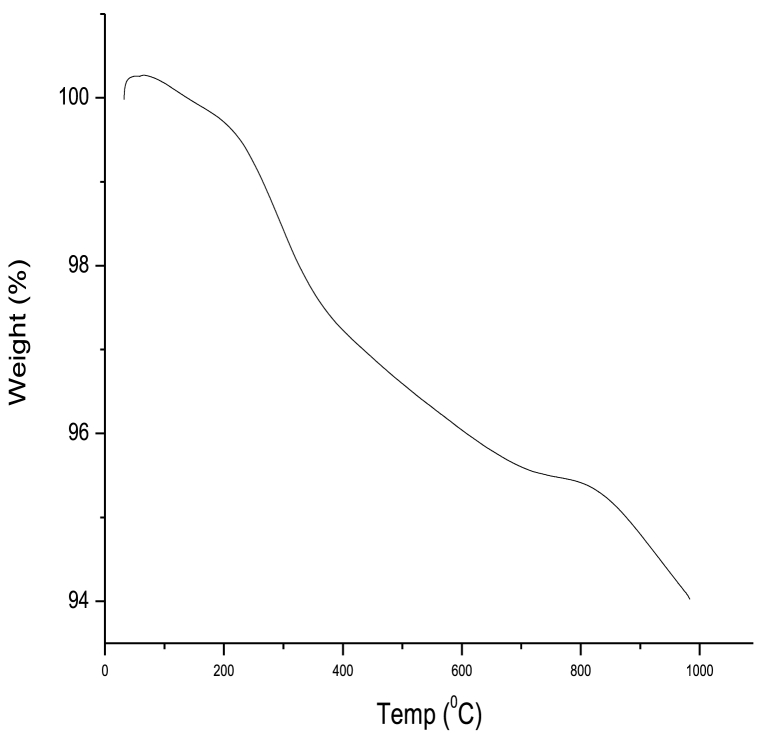
Thermograph of the Ag/MgO nanoparticles sample. The thermogravimetric analysis confirmed the phase purity analysis of samples through thermal treatments.

Fig. 7 displays the thermograph of pure Ag/MgO nanoparticles, along with the mass loss determined through thermogravimetric analysis. The calcination of Ag/MgO nanoparticles in the temperature range of 24–430 °C led to the removal of physically adsorbed water and nitrogen oxide gases, as well as the conversion of Ag and MgO(OH)_2_ into Ag/MgO. This process highlights the importance of calcination in obtaining the desired phase and composition for the nanoparticles.

The second mass loss event occurs in the temperature range of approximately 430 °C–1000 °C. This mass loss is attributed to the transformation of any residual carbon into CO_2_ gas [43]. The elimination of carbon impurities is a critical step in ensuring the purity and performance of the Ag/MgO nanoparticles. The thermogravimetric analysis provides valuable insights into the thermal stability and decomposition processes of the nanoparticles, which are crucial for optimizing synthesis parameters and understanding their behaviour in various applications where elevated temperatures may be encountered [43].

#### 3.1.5 Hydrodynamic size and zeta potential

**Fig. 8:**
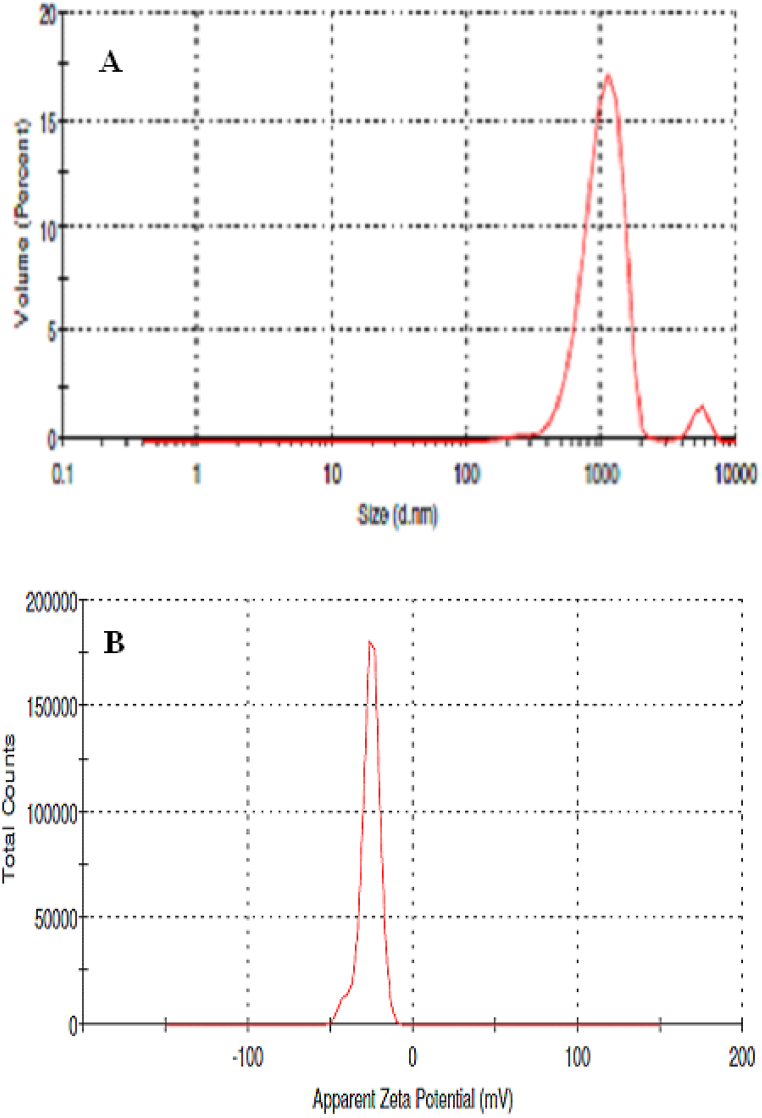
Characterization of Ag/MgO nanoparticles, depicting (A) particle size distribution using dynamic light scattering and (B) surface charge assessment through zeta potential measurements.

Fig. 8 presents the characterization of Ag/MgO nanoparticles in terms of their average hydrodynamic diameter, polydispersity index (PDI), and zeta potential. The average hydrodynamic diameter, which provides information on the particle size distribution in a suspension, was found to be 932.3 ± 22.0 nm (Fig. 8 section A). This relatively large size may be attributed to the aggregation behaviour of the nanoparticles, as discussed earlier.

The polydispersity index (PDI), a measure of the size distribution uniformity within the sample, was determined to be 0.4384 ± 0.0232. A PDI value close to 0 indicates a highly uniform size distribution, while a value closer to 1 implies a broad size distribution. In this case, the PDI suggests a moderately uniform distribution of particle sizes within the sample.

Additionally, the zeta potential of the Ag/MgO nanoparticles was measured to be −26.1 ± 0.6 mV (Fig. 8 section B). The zeta potential is an indicator of the surface charge and colloidal stability of the nanoparticles in suspension. A high absolute value of zeta potential typically implies better stability and lower aggregation tendencies. In this case, the moderately negative zeta potential value suggests that the particles may still be prone to aggregation, potentially affecting their performance in specific applications.

Overall, the information provided in Fig. 8 is crucial for understanding the behaviour of Ag/MgO nanoparticles in suspension, which can be important for various applications such as drug delivery, catalysis, and environmental remediation.

### 3.2 Anticancer effect of Ag/MgO nanoparticles

#### 3.2.1 Cytotoxicity

**Fig. 9:**
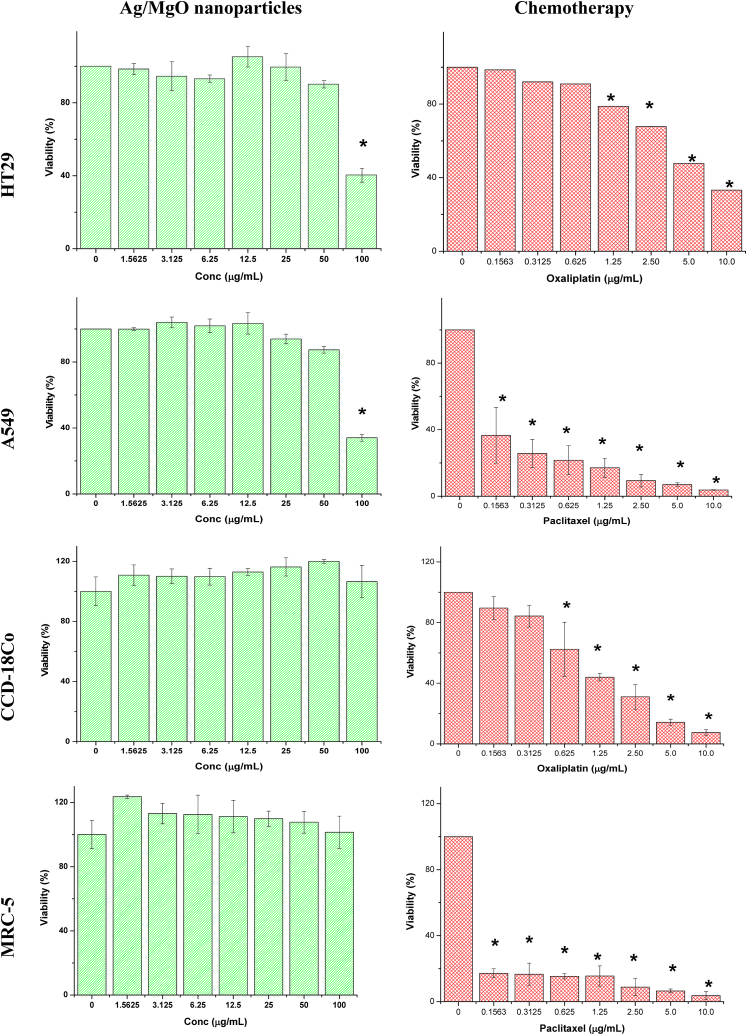
Viability of normal and cancer cells treated with Ag/MgO nanoparticles and chemotherapeutics as determined by the 4,5-dimethylthiazol-2-yl)-2,5-diphenyltetrazolium bromide assay. HT29 = Human colorectal adenocarcinoma cell line; A549 = Human lung adenocarcinoma; CCD-18Co = human normal colorectal cell line; MRC-5 = human normal lung cells. Zero concentration is for the nontreated cells. Values are presented as mean ± standard deviation (n = 3 wells/treatment). *Significantly significant value (*p* < 0.05) compared with the nontreated cells.

Following a 24-h exposure, a 100 μg/mL Ag/MgO nanoparticle suspension resulted in 60% growth inhibition of HT29 cells, whereas a 50 μg/mL suspension led to a 10% inhibition (Fig. 9). In contrast, normal human colon CCD-18Co cells exhibited a relatively low susceptibility to the toxic effects of Ag/MgO nanoparticles. Among the cell lines, HT29 cells displayed the lowest IC_50_ value in response to Ag/MgO nanoparticles, indicating their heightened vulnerability to the antiproliferative effects of these nanoparticles (Table 1). A 100 μg/mL Ag/MgO nanoparticle suspension, after a 24-h treatment, induced 66% cell death in A549 cells, while a 50 μg/mL suspension caused 13% cell death. Under similar treatment conditions, the nanoparticles did not demonstrate a significant antiproliferative effect (p > 0.05) on normal lung MRC-5 cells.

**Table 1:** IC_50_ values of Ag/MgO nanoparticles, oxaliplatin, and paclitaxel on human colorectal (HT29) and lung (A549) adenocarcinoma, and normal (CCD-18Co) and lung (MRC-5) cell lines after 24 h of treatment.

Treatment	IC_50_ (μg/mL)			
	HT29	A549	CCD-18Co	MRC-5
Ag/MgO	90.2 ± 2.6	85.0 ± 3.5	–	–
Oxaliplatin	4.7 ± 0.0	–	10.5 ± 8.1	–
Paclitaxel	–	0.12 ± 0.1	–	0.8 ± 1.6

As indicated by the low IC_50_ value, oxaliplatin exhibits high toxicity to HT29 and CCD-18Co cells, while paclitaxel is toxic to A549 and MRC-5 cells. The antiproliferative effects of these chemotherapeutic agents were more pronounced on cancer cells compared to normal cells.

The IC_50_ value of Ag/MgO nanoparticles on HT29 cells was roughly 19 times higher than that of the commercial anti-colorectal cancer drug oxaliplatin, and approximately 700 times higher on A549 cells compared to the commercial anticancer drug paclitaxel.

#### 3.2.2 Caspase-3, -8 and -9

After administering Ag/MgO nanoparticles to A549 and HT-29 cells for 12 and 24-h treatment periods, a noticeable increase in caspase activity was detected for both cell types at all tested concentrations. Caspase activity is associated with the initiation and execution of apoptosis, or programmed cell death, which is a key mechanism in controlling cell proliferation and maintaining tissue homeostasis. The increased caspase activity in A549 and HT-29 cells in response to Ag/MgO nanoparticle treatment suggests that these nanoparticles may trigger apoptosis, leading to the inhibition of cell growth or even cell death. This observation highlights the potential of Ag/MgO nanoparticles for therapeutic applications, specifically in the context of cancer treatment, as they appear to influence the apoptotic pathways in cancer cells.

##### 3.2.2.1 Caspase-3

**Fig. 10:**
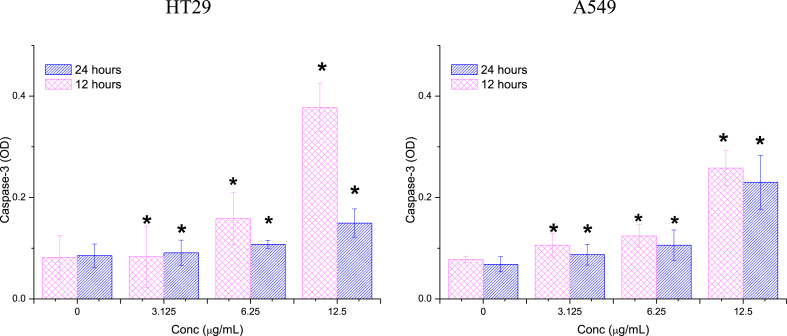
Caspase-3 activities in the human colorectal (HT29) and lung adenocarcinoma (A549) cells treated with Ag/MgO nanoparticles for 12 and 24 h. Zero concentration is for the nontreated cells. Values are presented as mean ± standard deviation (n = 3 wells/treatment). *Significantly significant value (*p* < 0.05) compared with the nontreated cells.

Caspase-3 activity in HT29 cells exhibited a substantial and statistically significant (p < 0.05) increase following exposure to 6.25 and 12.5 μg/mL Ag/MgO nanoparticles for 12 h (Fig. 10). However, the enzyme's activity diminished after 24 h, indicating that the impact of Ag/MgO nanoparticles on HT29 cells may not be time dependent. In A549 cells, the rise in caspase-3 activity commenced at 3.125 μg/mL Ag/MgO nanoparticle treatment and continued to escalate as the treatment concentrations increased. In these cells, the influence of Ag/MgO nanoparticles on caspase-3 activity was more pronounced after 12 h of treatment compared to 24 h.

##### 3.2.2.2 Caspase-8

**Fig. 11:**
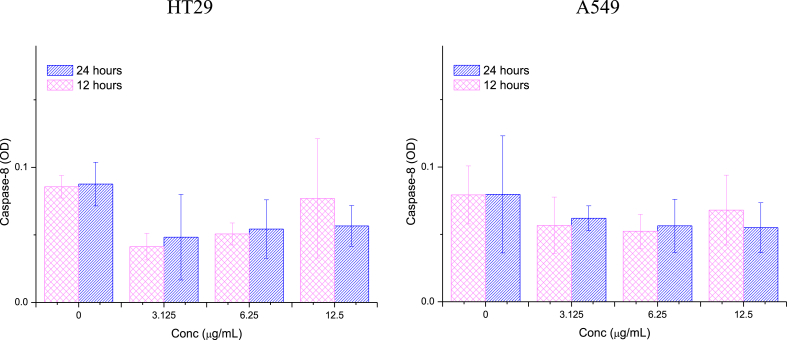
Caspase-8 activities in the human colorectal (HT29) and lung adenocarcinoma (A549) cells treated with Ag/MgO nanoparticles for 12 and 24 h. Zero concentration is for the nontreated cells. Values are presented as mean ± standard deviation (n = 3 wells/treatment). *Significantly significant value (*p* < 0.05) compared with the nontreated cells.

Intriguingly, the activity of caspase-8 in HT29 and A549 cells treated with Ag/MgO nanoparticles did not exhibit significant changes (p > 0.05) in relation to treatment dosage or duration of exposure. Nevertheless, caspase-8 activity was lower in the treated cells compared to the untreated cells (Fig. 11).

##### 3.2.2.3 Caspase-9

**Fig. 12:**
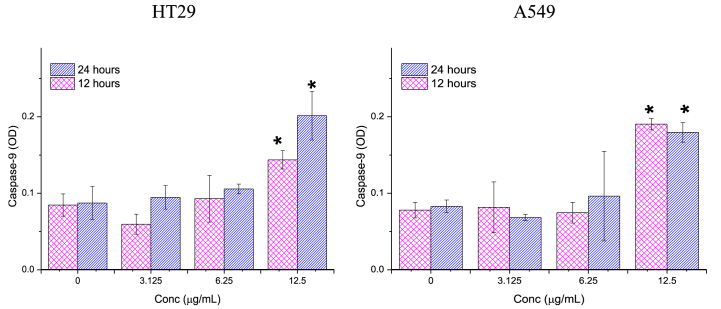
Caspase-9 activities in the human colorectal (HT29) and lung adenocarcinoma (A549) cells treated with Ag/MgO nanoparticles for 12 and 24 h. Zero concentration is for the nontreated cells. Values are presented as mean ± standard deviation (n = 3 wells/treatment). *Significantly significant value (*p* < 0.05) compared with the nontreated cells.

A significant increase (p < 0.05) in caspase-9 activity was observed in HT29 and A549 cells treated with 12.5 μg/mL Ag/MgO nanoparticles for both 12 and 24-h durations (Fig. 12). Notably, the impact of 12.5 μg/mL Ag/MgO nanoparticle treatment on caspase-9 activity in A549 cells was more pronounced after 12 h compared to 24 h of treatment.

#### 3.2.3 *Bcl-2*, *Bax*, *p53* and cytochrome C proteins

##### 3.2.3.1 Bcl-2

**Fig. 13:**
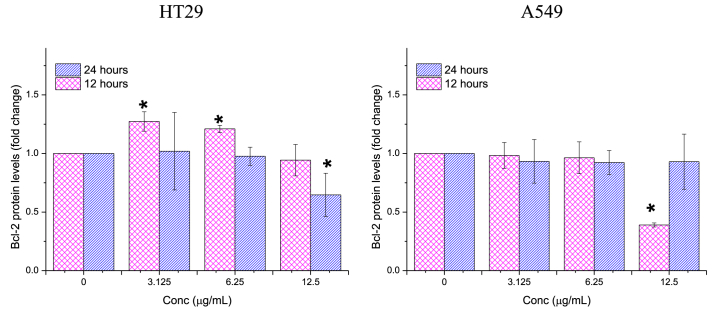
*Bcl-2* protein expression in the human colorectal (HT29) and lung adenocarcinoma (A549) cells treated with Ag/MgO nanoparticles for 12 and 24 h. Zero concentration is for the nontreated cells. Values are presented as mean ± standard deviation (n = 3 wells/treatment). *Significantly significant value (*p* < 0.05) compared with the nontreated cells.

The influence of Ag/MgO nanoparticles on the expression of the Bcl-2 protein in cancer cells was less apparent. Treatment with 12.5 μg/mL Ag/MgO nanoparticles resulted in the down-regulation of this protein only after 12 h in A549 cells and 24 h in HT29 cells (Fig. 13).

##### 3.2.3.2 Bax

**Fig. 14:**
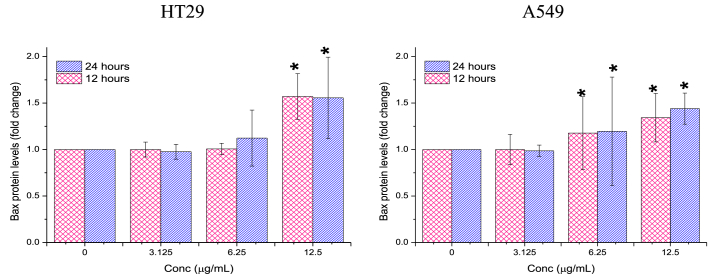
*Bax* protein expression in the human colorectal (HT29) and lung adenocarcinoma (A549) cells treated with Ag/MgO nanoparticles for 12 and 24 h. Zero concentration is for the nontreated cells. Values are presented as mean ± standard deviation (n = 3 wells/treatment). *Significantly significant value (*p* < 0.05) compared with the nontreated cells.

Ag/MgO nanoparticles exhibited a more pronounced impact on the up-regulation of Bax protein in A549 cells compared to HT29 cells. In A549 cells, the elevation of Bax protein expression began after treatment with 6.25 μg/mL Ag/MgO nanoparticles. In contrast, the increase in Bax protein expression in HT29 cells was only observed following treatment with 12.5 μg/mL Ag/MgO nanoparticles (Fig. 14).

##### 3.2.3.3 p53

**Fig. 15:**
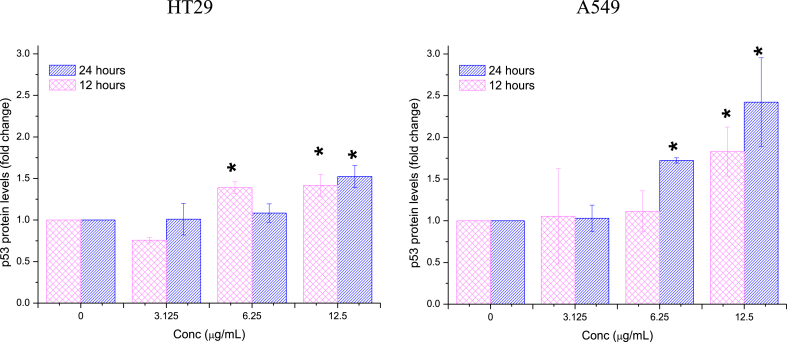
*p53* protein expression in the human colorectal (HT29) and lung adenocarcinoma (A549) cells treated with Ag/MgO nanoparticles for 12 and 24 h. Zero concentration is for the nontreated cells. Values are presented as mean ± standard deviation (n = 3 wells/treatment). *Significantly significant value (*p* < 0.05) compared with the nontreated cells.

Fig. 15 displays the expression of the p53 protein in HT29 and A549 cells after treatment with Ag/MgO nanoparticles. The expression of p53 in cancer cells began to significantly increase (p < 0.05) following treatment with 6.25 μg/mL Ag/MgO nanoparticles. The impact of the nanoparticles on the p53 protein expression in these cell lines was largely comparable, with the exception that the expression of p53 protein rose after 12 h in HT29 cells and after 24 h in A549 cells at the 6.25 μg/mL treatment concentration. When treated with 12.5 μg/mL Ag/MgO nanoparticles, the increase in p53 protein expression was similar for both cell lines.

##### 3.2.3.4 Cytochrome C

**Fig. 16:**
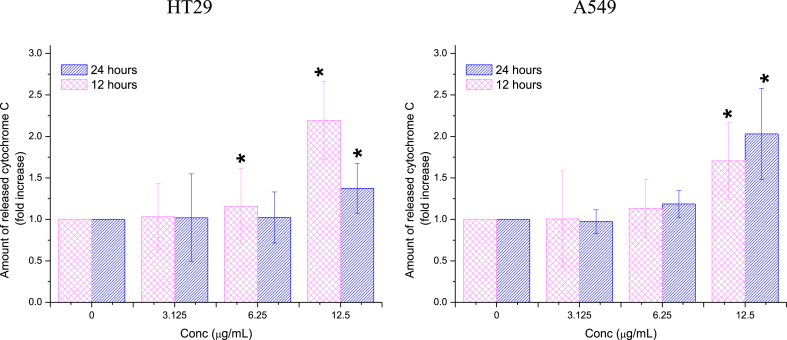
Cytochrome C release in the human colorectal (HT29) and lung adenocarcinoma (A549) cells treated with Ag/MgO nanoparticles for 12 and 24 h. Zero concentration is for the nontreated cells. Values are presented as mean ± standard deviation (n = 3 wells/treatment). *Significantly significant value (*p* < 0.05) compared with the nontreated cells.

Ag/MgO nanoparticles induced significant elevations (P < 0.05) in cytochrome C expression in A549 cells solely at a 12.5 μg/mL treatment concentration (Fig. 16). In HT29 cells treated with Ag/MgO nanoparticles, cytochrome C expression was higher after 12 h of treatment compared to 24 h, whereas in A549 cells, the opposite trend was observed, with cytochrome C expression being higher after 24 h than after 12 h of treatment.

#### 3.2.4 Morphological examination

**Fig. 17:**
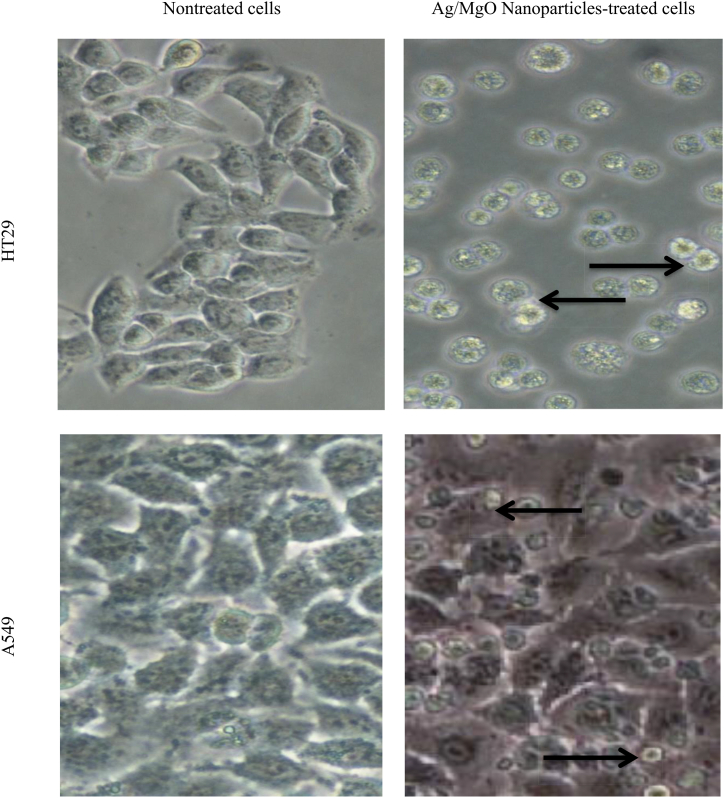
Morphology of the human colorectal (HT29) and lung adenocarcinoma (A549) cells treated with Ag/MgO nanoparticles for 24 h. Apoptotic cells show typical membrane blebbing, loss of membrane integrity, and shrinkage (black arrow). Mag. 200×.

When exposed to their respective IC_50_ concentrations of Ag/MgO nanoparticles for a duration of 24 h, both HT29 and A549 cells experienced considerable morphological transformations (Fig. 17). The cancer cells that underwent treatment displayed characteristics such as cell detachment from the surrounding matrix, shrinkage in cell size, and the formation of membrane blebs. It is noteworthy that these morphological changes were more visibly evident in the HT29 cells compared to the A549 cells, indicating a potential difference in their responses to Ag/MgO nanoparticle treatment.

## 4 Discussion

The nanoparticles can still be synthesized in different ways and with varying size ranges (eg: 10–100 nm); these characteristics being important for the properties they present [44,45]. Silver nanoparticles have excellent antibacterial properties and according to many environmental protection agencies, majority of registered silver-based biocidal products are likely contain silver nanoparticles [[46], [47], [48]]. However, just as silver nanoparticles can have antibacterial activity, they can also cause neurological and embryonic damage [49].

The design and development of new nanoparticles has been of fundamental importance for the medicine, which explores the new physicochemical properties of these materials in a multitude of new articles and products [50]. However, the nanometric size of these particles combined with their physicochemical characteristics raise concerns about their biological effects, which may represent risks to health and the environment [50,51].

The TEM and SEM analyses showed that the Ag/MgO nanoparticles were predominantly cuboidal and crystalline in structure. These findings support similar observations in a previous study using the XRD pattern [52]. The ultrastructural analysis showed that the Ag/MgO nanoparticles have a nanosized distribution of 31–68 nm. However, when we employed the XRD and TEM analyses, the size range of the Ag/MgO nanoparticles was between 39 and 48 nm. The Ag/MgO nanoparticles that we developed were larger than the silver nanoparticles developed by Zuberek et al., 2015 [52], where they reported that the average diameter of silver nanoparticles was 20.0 nm.

EDX was used in the elemental analysis of the Ag/MgOnanoparticles, The silver component of the nanoparticles was less than 1%. The low silver content in the Ag/MgO nanoparticles is suggested to be due to the incomplete precipitation of the magnesium metal precursors during the co-precipitation process [43].

The BET surface area of the Ag/MgOnanoparticles was 14.78 m^2^/g. The BET surface area of the Ag/MgO nanoparticles is considerably lower than the 23.81 m^2^/g reported by another study on silver nanoparticles prepared from bulk metal silver [53]. It is suggested that the low BET surface area of the Ag/MgO nanoparticles is due to magnesia pores being partially covered by a layer of silver particles [54]. However, the BET surface area of the MgO was almost similar to the conventional silver nanoparticles with binary support [43]. The cuboidal structure also provides the Ag/MgO nanoparticles with specific features including extremely low metal dispersion and small surface areas. The low metal dispersion and small surface areas may be the result of strong interactions between the silver layer and the supporting MgO [43].

The Ag/MgO nanoparticles were shown to exhibit in vitro anti-cancer properties, particularly on the colorectal and lung cancer cells [33,[55], [56], [57], [58], [59], [60], [61]]. Silver nanoparticles kill these cancer cell lines while remaining relatively innocuous to non-cancerous cells. When administered intratumorally with concomitant infrared treatment, the silver nanoparticles significantly inhibited tumor growth [[62], [63], [64]]. A similar cytotoxic effect of silver nanoparticles was observed in Dalton’s lymphoma ascites [65], lung [59], and oral cancer [66] cells. While the mechanism of silver nanoparticle cancer cell toxicity is not fully understood, it is believed that among the mechanisms for the anti-cancer effects of Ag/MgO nanoparticles is the oxidative damage caused by the silver ions released from the nanoparticles [67,68]. Cancer treatment, despite the great therapeutic advance, still generates many side effects to the patient and has drug multidrug resistance [69,70]. Science has constantly sought innovations to overcome these obstacles, improving treatment efficiency and generating fewer adverse effects [69,70]. Treatment with nanoparticles can be an effective alternative for the treatment of cancer, and because of this, several studies are looking for answers [8,9].

The selectivity index of nanoparticles is calculated as the average of their IC_50_ value on the normal cells divided by their IC_50_ value on the cancer cells [71]. The toxic concentration of the *ideal anticancer compound* should be *relatively* high while the active concentration should be *very low* [69], thus, a high selectivity index. We showed that the average selective index of Ag/MgO nanoparticles for the human colorectal HT29 and lung A549 cancer cells using the normal colorectal CCD-18Co and lung MRC-5 cells as the numerator was 3.83 and 4.99, respectively. Based on these values, the Ag/MgO nanoparticles have greater selectivity for the lung than colorectal cancer cells in the exertion of anti-cancer effects [69,70].

Caspase-9 and -8 are the mediators of the mitochondria-mediated (intrinsic) and receptor-mediated (extrinsic) apoptotic pathways, respectively. Caspase-3 is another major enzymatic marker responsible for the upstream activation of apoptosis [72,73]. In this study, the activities of caspase-3 and -9 were significantly higher (*p* < 0.05) in the Ag/MgO nanoparticle-treated than the nontreated HT29 and A549 cells. However, the Ag/MgO nanoparticles did not significantly affect caspase-8 activity in the cancer cells. Thus, it can be concluded that the cancer cell antiproliferative effect of Ag/MgO nanoparticles occurs, at least partially, via the intrinsic pathway of apoptosis.

The Ag/MgO nanoparticles were toxic to HT29 and A549 cells only at high concentrations. We investigated the apoptotic effect of the Ag/MgO nanoparticles by determining the expression of *Bcl-2, Bax, p53*, and cytochrome C proteins in the treated cancer cells. Ag/MgO nanoparticles appeared to down-regulate the anti-apoptotic *Bcl-2* and up-regulated the pro-apoptotic *Bax* protein expressions. At the same time, the nanoparticles, at high treatment concentrations, up regulated the tumor suppressor protein, *p53*, and increased cytochrome C levels (in the cytoplasm) in the HT29 and A549 cells.

Silver-containing nanoparticles were shown to be toxic to cancer cells including gastric adenocarcinoma (AGS) [74], cervical cancer (HeLa) [75], prostate cancer (PC3) [76], colon cancer (HCT116) [77], and two human hormone-dependent breast cancer, T47D [78,79] and MCF-7 cells [80]. The flow cytometry analysis showed that treatment with silver-containing nanoparticles caused the T47D cell population to shift from the viable to the apoptotic phase [79]. In the AGS cells, silver nanoparticle-containing drugs induced apoptosis via the *p53*, *Bax/BCL-2*, and caspase pathways [74].

We showed that the Ag/MgO nanoparticles are antiproliferative toward human colorectal and lung cancer cells. The mechanism of cell death induced by the Ag/MgO nanoparticles is through apoptosis, involving the mitochondrial pathway, cytochrome C release, and modification in anti- and pro-apoptotic protein expressions. The antiproliferative effect of Ag/MgO nanoparticles was also evident with the development of typical apoptotic morphology in the treated cancer cells. Although the Ag/MgO nanoparticles showed anticancer activities, the effect was not as potent as or comparable with that of known anticancer therapeutics, oxaliplatin, and paclitaxel.

This study contributes to the understanding of the toxicity mechanisms of silver nanoparticles in the sense of providing support for their action in the induction of apoptosis, due to the greater toxicity observed in the HT29 and A549 cells when compared to with the CCD-18Co and MRC-5 cells Furthermore, the analysis of nucleoplasmic bridges (NPBs), which carried out in the previous work [81], will make a pioneering contribution to toxicological studies with silver nanoparticles. However, future studies should be carried out in order to investigate in detail the changes in DNA and study the dynamics of DNA repair mechanisms after treatment with silver nanoparticles, palladium nanoparticles and platinum nanoparticles at different concentrations.

## 5 Conclusion

In this study, the Ag/MgO nanoparticles that were synthesized exhibited a nanoscale size distribution between 31 and 68 nm, averaging at 43.5 ± 10.6 nm. Through in vitro cytotoxicity assays, it was demonstrated that these nanoparticles possessed a toxic effect on human colorectal and lung cancer cells, while maintaining their non-toxic nature towards normal colorectal CCD-18Co and lung MRC-5 cells. The antiproliferative properties of the Ag/MgO nanoparticles were found to be mediated by apoptosis involving the caspase-3 and caspase-9-dependent mitochondrial signaling pathway. This apoptotic process was accompanied by a down-regulation of the anti-apoptotic protein Bcl-2 and an up-regulation of the pro-apoptotic protein Bax.

Furthermore, the Ag/MgO nanoparticles were observed to stimulate the release of mitochondrial cytochrome C, which plays a crucial role in cell apoptosis. In addition, these nanoparticles were found to enhance the expression of the p53 tumor suppressor protein, a key regulatory protein involved in cell cycle control and apoptosis. Consequently, this study underscores the potential of Ag/MgO nanoparticles to be developed into an effective and targeted anti-cancer and anti-tumor therapeutic agent, offering a promising avenue for further research and development in cancer treatment.

## Author contribution statement

Mohamed Al-Fahdawi; Mothanna Al-Qubaisi: Performed the experiments; Wrote the paper.

Ahmed Faris Aldoghachi; Fatah Alhassan: Analyzed and interpreted the data.

Faris Al-Doghachi: Performed the experiments.

Hussah Alshwyeh: Contributed reagents, materials, analysis tools or data; Wrote the paper.

Abdullah Rasedee: Conceived and designed the experiments; Wrote the paper.

Sulaiman Mohammed Alnasser; Wisam Nabeel Ibrahim: Conceived and designed the experiments.

## Funding statement

This research did not receive any specific grant from funding agencies in the public, commercial, or not-for-profit sectors.

## Data availability statement

Data included in article/supplementary material/referenced in article.

## Declaration of interest’s statement.

The authors declare no conflict of interest.

## Funding

The publication of this article was funded by the Qatar National Library.

## Declaration of competing interest

The authors declare that they have no known competing financial interests or personal relationships that could have appeared to influence the work reported in this paper.
